# The Influence of Corn Straw Extrusion Pretreatment Parameters on Methane Fermentation Performance

**DOI:** 10.3390/ma13133003

**Published:** 2020-07-06

**Authors:** Karol Kupryaniuk, Tomasz Oniszczuk, Maciej Combrzyński, Wojciech Czekała, Arkadiusz Matwijczuk

**Affiliations:** 1 Department of Thermal Technology and Food Process Engineering, University of Life Sciences in Lublin, Głęboka 31, 20-612 Lublin, Poland; karol.kupryaniuk@o2.pl; 2 Institute of Biosystems Engineering, Poznań University of Life Sciences, Wojska Polskiego 50, 60-627 Poznań, Poland; wojciech.czekala@up.poznan.pl; 3 Department of Physics, Lublin University of Life Sciences Akademicka 13, 20-950 Lublin, Poland

**Keywords:** corn straw, extrusion-cooking, pretreatment biogas production, renewable energy, FTIR spectra

## Abstract

The aim of the study is to determine the energy consumption of the extrusion-cooking process of corn straw under various conditions (screw speed, moisture content), water absorption measurements and water solubility indices as well as biogas efficiency evaluation. The extrusion-cooking of corn straw was carried out using a single screw extruder with L/D = 16:1 at various rotational screw speeds (70, 90, and 110 rpm) and with various initial moisture content of raw material (25 and 40%). Prior to the process, the moisture content of the raw material was measured, and next, it was moistened to 25 and 40% of dry matter. For example, at 70 rpm extruder screw speed, the temperature range was 126–150 °C. Energy consumption of straw pretreatment through extrusion-cooking was assessed in order to evaluate the possibility of using the process in an agricultural biogas plant. Biogas and methane efficiency of substrates after extrusion was tested in a laboratory scale biogas plant and expressed as a volume of cumulative methane production for fresh matter, dry matter, and dry organic matter. Pretreated corn straw moistened to 25% and processed at 110 rpm during the extrusion-cooking processing produced the most advantageous effect for methane and biogas production (51.63%) efficiency as compared to corn straw without pretreatment (49.57%). Rotational speed of the extruder screw influenced biogas and methane production. With both dry matter and dry organic matter, the increase of rotational speed of the extruder screw improved the production of cumulated biogas and methane. Pretreatment of corn straw has a positive effect on the acquisition of cumulated methane (226.3 Nm^3^ Mg^−1^ for fresh matter, 243.99 Nm^3^ Mg^−1^ for dry matter, and 254.83 Nm^3^ Mg^−1^ for dry organic matter). Preliminary analysis of infrared spectra revealed changes in the samples also at the molecular level, thus opening up the possibility of identifying marker bands that account for specific degradation changes.

## 1. Introduction

The most common substrates used in biogas plants are corn silage, slurry, and feedstocks in the form of lignocellulosic biomass. Triticale, maize/corn, rape straw, and hay also find application in such installations. Corn silage is one of the most widespread substrates of vegetable origin used for co-fermentation in agricultural biogas plants. In comparison to other cereal plants, it has a higher dry matter yield per hectare of crop, higher biogas yield, and lower cultivation costs. The methane fermentation process carried out in biogas plants helps dispose of waste substances that are seen as a burden to ecosystems. Waste from livestock production exhibits proper composition for the purpose of methane production. This is, for example, liquid manure, manure or droppings from poultry production. Much less desirable raw materials for biogas production are wastes with a high lignin content. Lignocellulosic biomass contains natural polymers such as cellulose, hemicellulose, and lignin [1,2,3]. Cellulose and hemicellulose as carbohydrates suitable as feedstock are fermentable after hydrolysis for bioenergy production. Unfortunately, lignocellulosic biomass is resistant to biodegradation by enzymes and microbes because of its inherent characteristics [4,5,6]. According to Eastman and Ferguson [7], the hydrolysis of sparingly soluble polymers, such as cellulose, lignins, and also decomposable fat, protein, and carbohydrate, is a limiting step in the rate of fermentation. They found that carbohydrates were more readily degraded than nitrogenous materials, and lipids remained unaffected. As shown by Di Matteo [8] scale becomes the crucial challenge to associate the feasible energy production due to application of cooking wastes in cities as effective substrate in biogas production. Energy balance is affected by the biogas production plant layout, especially by the choice of the digester technology. The whole chain calculations could be possible if industrial grade processes were tested as different equipments are needed for the treatment of food and agricultural wastes. Generally, any type of biomass can be used as a substrate for biogas production, provided it contains carbohydrates, proteins, fats, cellulose, and hemicellulose as the main components. Ultimately, this process must be preceded by earlier treatment that will disintegrate lignin, as well as releasing hemicellulose and cellulose. Pretreatment enhances the fermentation of substrates, which increases the efficiency of biogas production, including methane [9]. Scientific sources confirm that the use of pretreatment can improve the efficiency of biogas production to over 90% for raw materials such as grass, corn straw, and wood [6,10].

There are several methods of pretreatment of lignocellulosic biomass, among them chemical, biological, mechanical, and pressure-thermal treatment. Chemical treatment, useful in increasing methane efficiency, could be based on oxidation or treatment with peroxides, alkalis, and acids. In biological treatment methods, fungi, microbes, or enzymes are responsible for silage making. It is also worth mentioning that biological pretreatment methods are not only used for the production of silage itself but also microbes or enzymes can be added directly to the ready-made raw material. Disintegration of lignin, hemicellulose, and cellulose by mechanical methods include, i.e., radiation by ultrasound or microwaves, disintegration, extrusion, or steam explosion. All these methods are aimed to digest lignocellulosic substrates to increase biogas and methane efficiency during conversion to biogas by anaerobic digestion [6,11,12,13].

Extrusion-cooking is a pressure and thermal process that uses shear forces acting on processed material. The material undergoes heating and plasticization, so the mass can be shaped by a nozzle and expands [14,15,16]. The formation of shear forces and pressure is caused by the rotation speed of extruder screws. Single-screw and twin-screw extruders are used for the extrusion-cooking process. During the process, the extruder chamber has a high temperature reaching 200 °C, and various pressures can be obtained depending on the unit configuration. The process time during which biomass stays inside the extruder chamber depends on the length of the plasticizing system and the rotational speed of the extruder screw. During the processing of plant raw materials, lignin is broken in biomass; cellulose and hemicelluloses are also released and facilitate conversion [10,17].

Agricultural biogas plants based on the anaerobic digestion process implemented on a large scale around the world, use biomass obtained from plantations of energy plants as well as by-products and wastes of plant and animal origin. The NaWaRo biogas production system (NachWachsendeRohstoffe) mainly uses silage from plants (beet, corn, grass, etc.,). Other substrates, such as slurry, agricultural waste or grain, are also used depending on the specific circumstances of the processing farm. Substrates such as grass, bark, straw, and hay can also be used to produce biopolymers [18,19,20]. The processing of biogas by-products and waste products is important for environmental protection and generates energy. In economic terms, biogas plants are the most profitable. This statement is related to the fact that in Poland, subsidies available for electric energy production from agricultural biogas plants are the highest (over 170 EUR/MWh), in fact over twice as high as in the case of photovoltaics and wind mills. Taking into account other possible profits from biogas plant exploitation (heat and digested sold, payment from problematic biowaste for treatment) it has to be assumed that in Polish conditions biogas plants can be the most profitable renewable energy installations.

The production of biogas through anaerobic digestion offers significant advantages over other forms of bioenergy production [21]. It has been evaluated as one of the most energy-efficient and environmentally beneficial technologies for bioenergy production [22]. The process of methane fermentation depends to a large extent on the conditions, availability, and type of the substrate. Depending on the raw material used, the mixture of gases produced as the final product will have different contents of methane, carbon dioxide, and other components. It is possible to use agricultural by-products: cereal straw, corn and cotton waste, plant stalks, animal residues (slurry, liquid manure, chicken poultry, litter), and others [23,24,25].

The aim of the study is to determine the stability of the extrusion-cooking process of lignocellulosic materials as well as performing the measurements of liquefaction of extrudates in water, the measurements of water absorption index and testing biogas efficiency. In addition, Fourier transform infrared spectroscopy (FTIR) was used to check selected samples for changes at the molecular level and attempt to identify marker bands of changes in the sample structures. The tests were carried out on plant extrudates produced at three rotational speeds of the extruder screw (70, 90 and 110 rpm) from raw material mixtures with the initial moisture of 25 and 40% of dry matter. It is noteworthy that the value the presented study stems from the evidence for the possibility of using straw—a cheap and readily available waste material generate in agricultural production, in the processes of extrusion and effective production of biogas (1 ton of straw can replace 3 tons of silage—a popular raw material used in biogas plants—while the prices of the two materials remain similar).

## 2. Materials and Methods

### 2.1. Materials

Corn straw used for research was harvested in 2017 and 2018 using a combine harvester and sifted to remove impurities. The straw was ground with a hammer shredder for a particle size of less than 10 mm and moistened to two moisture contents 25 and 40% by spraying with a suitable amount of water (extreme moisture levels at which the extrusion process can proceed without problems). Before processing, the materials were stored in sealed bags for a period of 24 h to unify the migration of water. The study pertained to maize straw whose chemical composition was derived from the most up-to-date source data [26,27]

### 2.2. Processing

**Figure 1:**
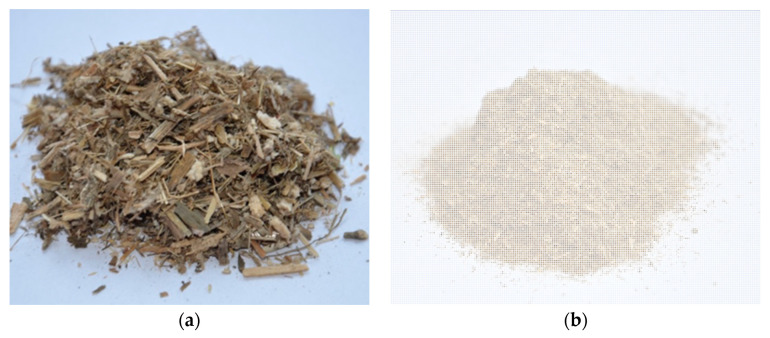
Raw (**a**) and extruded (**b**) corn straw used in the experiment.

The TS-45 (ZMChMetalchem, Gliwice, Poland) single-screw extruder with L/D = 16 and a 10 kW motor was used for the tests. The treatment processes were carried out in the temperature range 100–140 °C at three screw speeds of the 70, 90, and 110 rpm. The raw and treated materials are shown in Figure 1.

### 2.3. Energy Consumption during Processing

Energy consumption, expressed as SME [28] (specific mechanical energy), was calculated taking into account the engine load, the extruder-cooker working parameters, and the process efficiency of each test. The following formula was used:

(1) $$SME=\frac{n}{n_{m}}\times \frac{O}{100}\times \frac{P}{Q}\;\left({\mathrm{kWh}\;\mathrm{kg}}^{-1}\right)$$

where: *SME*—specific mechanical energy consumption $\left({\mathrm{kWh}\;\mathrm{kg}}^{-1}\right)$, *n*—screw rotation (rpm), *n_m_*—screw rating rotation (rpm), *O*—engine load compared to maximum (%), *P*—rated power (kW), and *Q*—process efficiency (kg h^−1^)

### 2.4. Water Absorption and Solubility Measurements

The water absorption index (WAI) and the water solubility index (WSI) were used to evaluate the cooking intensity by extrusion according to the method described by Bouasla et al. [29]. For this purpose, 7 mL of distilled water was added to 0.7 g of the extruded sample. The sample was mixed and put aside for 10 min, then centrifuged for 10 min at 15,000 rpm. The centrifuged samples were filtered, and the remains after gel formation were dried in an air oven at 105 °C until they were completely dry. The WAI was calculated as the mass of sludge per constant dry weight of the sample, and the WSI was calculated as the amount of residue dissolved in the supernatant. All measurements were made in triplicate.

### 2.5. Biogas Efficiency Analysis

**Scheme 1:**
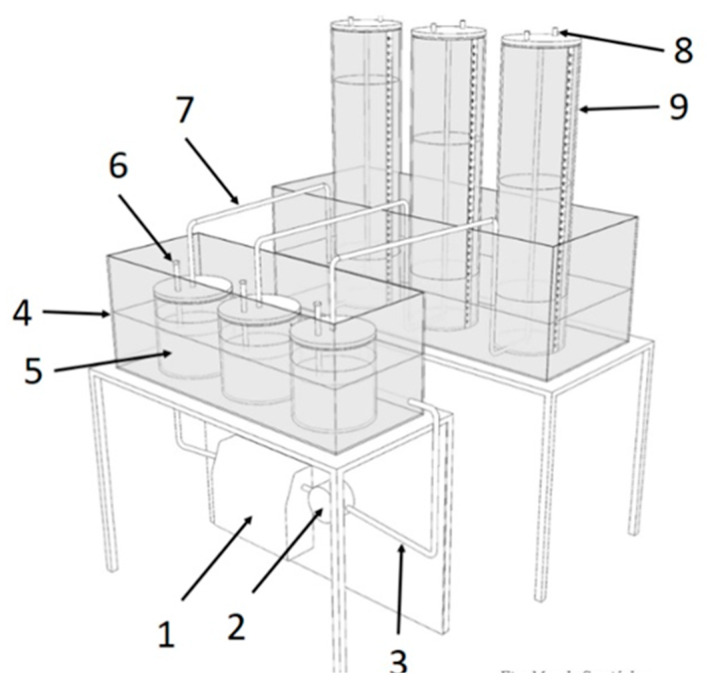
A design of biofermentor for biogas production research (3-chamber section): 1—water heater with temperature regulator, 2—water pump, 3—insulated conductors of calefaction liquid, 4—water coat, 5—biofermentor with the charge capacity of 2 dm^3^, 6—sampling tubes, 7—biogas transporting tube, 8—gas sampling valve, 9—biogas volume-scale reservoir [31].

The samples of extruded corn straw were analyzed for biogas efficiency using standard methods (DIN 38414/S8 and VDI 4630). Biogas production was carried out in a multi-chamber biofermentors at the Ecotechnologies Laboratory of the Institute of Biosystems Engineering, Poznań University of Life Sciences (Scheme 1) [30,31]. Fermentation reactors with a capacity of 2 dm^3^ were filled with inoculum (separated liquid fraction of digestate from real scale agricultural biogas) and extruded corn straw processed under different conditions. The percentage of dry matter in the inoculum should be between 1.5 and 2%. Before the experiment, the dry matter and the organic dry matter were measured, and the substrates were placed in an airtight fermentation reactor. The sample chambers were placed in temperature-controlled water (around 39 °C) which simulated the mesophilic conditions of the biogas plant. Biogas produced in each separate chamber was transferred to storage tanks filled with neutral liquid. The volume of produced biogas was measured every 24 h until the daily production of biogas was lower than 1% of the total volume of biogas produced. The test was performed in three replications. The biogas efficiency of the substrate (Nm^3^ Mg^−1^ organic dry matter) was calculated based on experimental results, as described by Dach et al. [30]. The normal volume of methane was calculated by multiplying the normal volume of dry gas by the methane content in dry gas and expressed as accumulated methane and combined biogas on dry matter and dry organic matter of corn straw.

### 2.6. Infrared Spectra Measurements

Measurements of infrared spectra for the analyzed samples were conducted with the use of a 670-IR spectrometer (Agilent, Santa Clara, CA, USA). An ATR (attenuated total reflection) attachment was used in the form of a ZnSe crystal with adequate geometry (truncated at 45°) to ensure 20-fold internal reflection of the absorbed beam. During the measurement, 24 scans were registered, and, subsequently, the programme averaged the results for all spectra. Prior to the measurement, the ZnSe crystal was cleaned using ultra-clear solvents by Sigma-Aldrich (Sigma-Aldrich, St. Louis, MO, USA). Prior to (1 h) and during the experiment, the measurement chamber was kept in an inert N_2_ atmosphere. Spectral measurements were recorded in the region from 550 to 3800 cm^−1^ at the resolution of 0.5 cm^−1^. The measurements were conducted at the Department of Biophysics Laboratory of the University of Life Sciences in Lublin. The spectra were analyzed and processed with the use of the Grams/AI software (version Suite 9.3) by ThermoGalactic Industries (Waltham, MA, USA). The spectra were normalized at the wave number of 1025 cm^−1^ (for easier analysis). All the spectra were measured at room temperature.

### 2.7. Statistical Analysis

The obtained results from three test replicates were subjected to a statistical analysis using Statistica 13.3 software (StatSoft,13.3, Cracow, Poland). Regression equations of quadratic models, correlation coefficients, and statistical one-way analysis of variance (ANOVA) with F-test and Kruskal-Wallis test were used to analyze the results at α = 0.05. The results were found as significantly affected by the screw speed at different moisture content as *p* value was lower than 0.05. Results are presented as mean value with ± standard deviation.

## 3. Results and Discussions

### 3.1. Effect of Processing Conditions on Extrusion Energy Consumption

**Figure 2:**
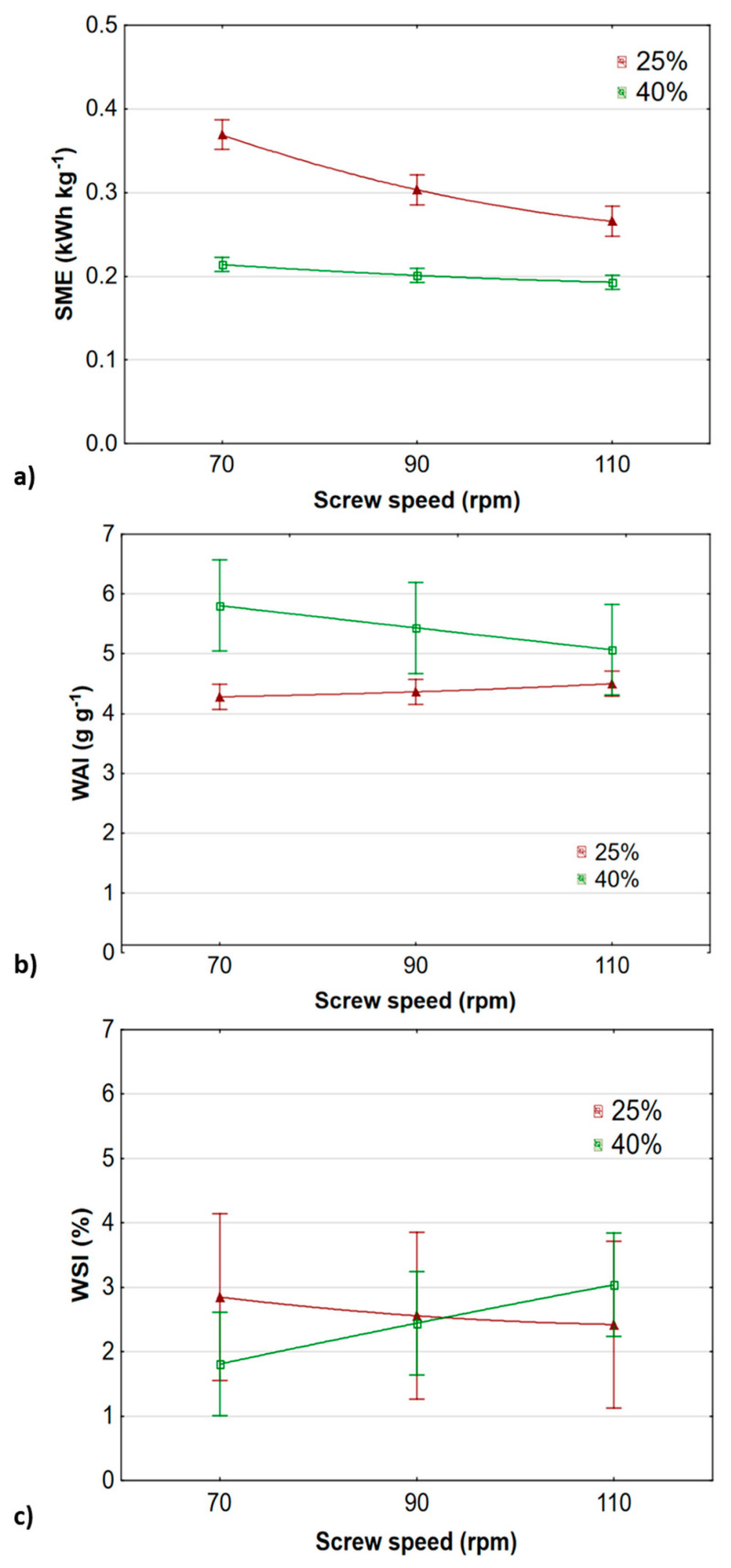
Selected properties of corn straw extruded under various screw speeds and moisture content: (**a**) energy consumption, (**b**) water absorption index, (**c**) water solubility index.

Pretreatment of corn straw by extrusion-cooking was carried out using a single-screw extruder type TS-45. The energy consumption during corn processing ranged from 0.206 to 0.332 kWh kg^−1^ at 70 rpm, from 0.202 to 0.301 kWh kg^−1^ at 90 rpm and from 0.198 to 0.269 kWh kg^−1^ at 110 rpm, as shown in Figure 2a. The moisture content increased from 25% to 40% reduces energy consumption, regardless of the rotational speed used during processing. Pre-processed samples of corn straw moistened to 40% were characterized by the lowest energy requirements during processing at 110 rpm, while samples processed at 25% of moisture content and 70 rpm were characterized by the highest energy demand. Water can act as a lubricant and reduce energy requirements, but it can also affect the mechanical behavior of materials obtained during extrusion cooking [32]. The increase in rotational speed of the extruder screw has an impact on the change of SD. A significant effect (*p* < 0.05) of screw speed on energy consumption was observed for the initial moisture content of both raw materials at the high values of correlation coefficients, which suggests a decrease in energy consumption along with a higher screw speed during processing (Table 1). A more intense effect was noted when 25% of moisture was used (higher values of F-test). These conditions generate the least energy demand during pretreatment, so it can be recommended as a pretreatment method useful for increasing biogas efficiency with low energy expenditure. Menardo et al. [33] used a counter-rotating twin-screw extruder driven by a 74 kW motor for the treatment of biomass containing rice straw silage, maize silage, and triticale silage. They reported that an increase in obtained energy for feeds containing 10% of rice straw was higher than the energy needed for extrusion, but the energy balance was close to zero when 30% of rice straw was used.

### 3.2. WAI and WSI of Extruded Corn Straw

The extruded corn straw was tested for the WAI and WSI, where the WAI reflects the amount of water bound by the product while the WSI represents the amount of small molecules dissolved in water depending on molecular damages caused by processing conditions [27,32]. The WAI and WSI results for extruded corn straw are shown in Figure 2b,c. For a moisture content of 25%, it was observed that the WAI increased with a higher screw rotational speed (Figure 2b). An opposite relationship was observed for the extruded corn straw with a moisture content of 40%. The increase in rotational speed of the extruder caused a gradual decrease in the value of WAI.

Water-soluble components also vary depending on the processing conditions. Small differences were observed for the WSI of corn straw extruded at 25% of moisture content regardless of the screw speed during processing (Figure 2c). For samples with the initial humidity of 40%, differences in the WSI were more significant. The highest WAI (5.80 g g^−1^) and the lowest WSI (1.81%) were recorded for samples processed at 40% humidity and 70 rpm. For the 25% moisture tests, the lowest WAI (4.28 g g^−1^) and the highest WSI (2.85%) were recorded for samples processed at 70 rpm. A significant effect (*p* < 0.05) of screw speed on the WSI was observed for 40% of initial moisture content of corn straw (Table 1). This was likely connected with the thermal and mechanical treatment during the extrusion-cooking of lignocellulose biomass, causing a larger active area as the processed material expanded by the pressure difference inside and outside of extruder barrel, and probably partial hydrolysis of cellulose components due to heating [10].

### 3.3. Biogas Efficiency

**Figure 3:**
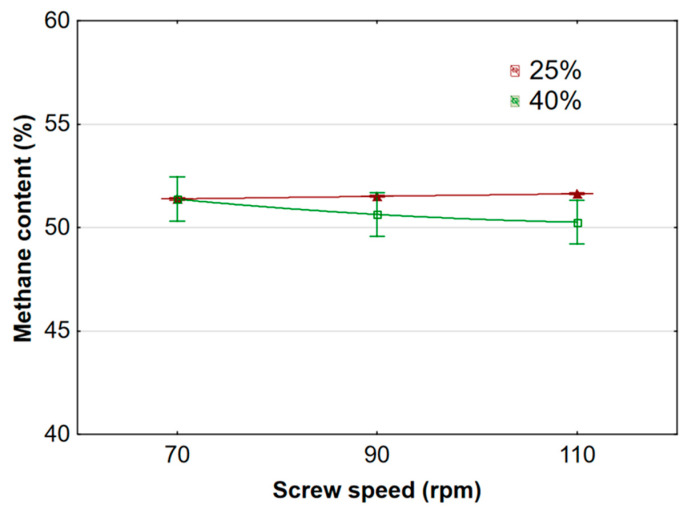
Methane content in produced biogas after fermentation of corn straw pretreated in various extrusion-cooking conditions.

The use of extrusion for pretreatment of corn straw used as a source of biomass affected significantly higher methane content (51.63% for pre-treated straw) after anaerobic fermentation compared to the control sample (49.57% for corn straw without pretreatment). Only the maximum methane efficiency, the substrate and the specific reaction rate must be known from a continuously digestion test [21,34,35,36]. The results regarding the methane content, as shown on Figure 3, indicated lower methane efficiency if corn straw was pretreated at 40% of initial moisture content. A significant effect (*p* < 0.05) of screw speed on methane content was observed if initial moisture content was of 25% with a high value of correlation coefficient (Table 1). Gizińska-Górna et al. [37] demonstrate that the content of methane in biogas ranged from 50.9 to 54.9% if common reed and Jerusalem artichoke were used for fermentation, and these values were rather lower compared to other typical substrates for biogas production. Pilarski et al. [10] found that pretreatment by extrusion was useful in improving the quantity of generated methane: as regards fresh matter for maize silage subjected to extrusion, the methane yield was 16.48% higher than that of the non-extruded silage. Application of a short single-screw extruder with L/D ratio of the screw of 6:1 and rotational speed of 200 rpm showed that maize straw silage after extrusion gave 35.30% more methane at mesophilic digestion than non-extruded material. Menardo et al. [33], using twin-screw extrusion of rice straw mixed with maize silage and triticale silage, reported an increase in the methane yield by 15.7% because organic matter degradation was started.

**Figure 4:**
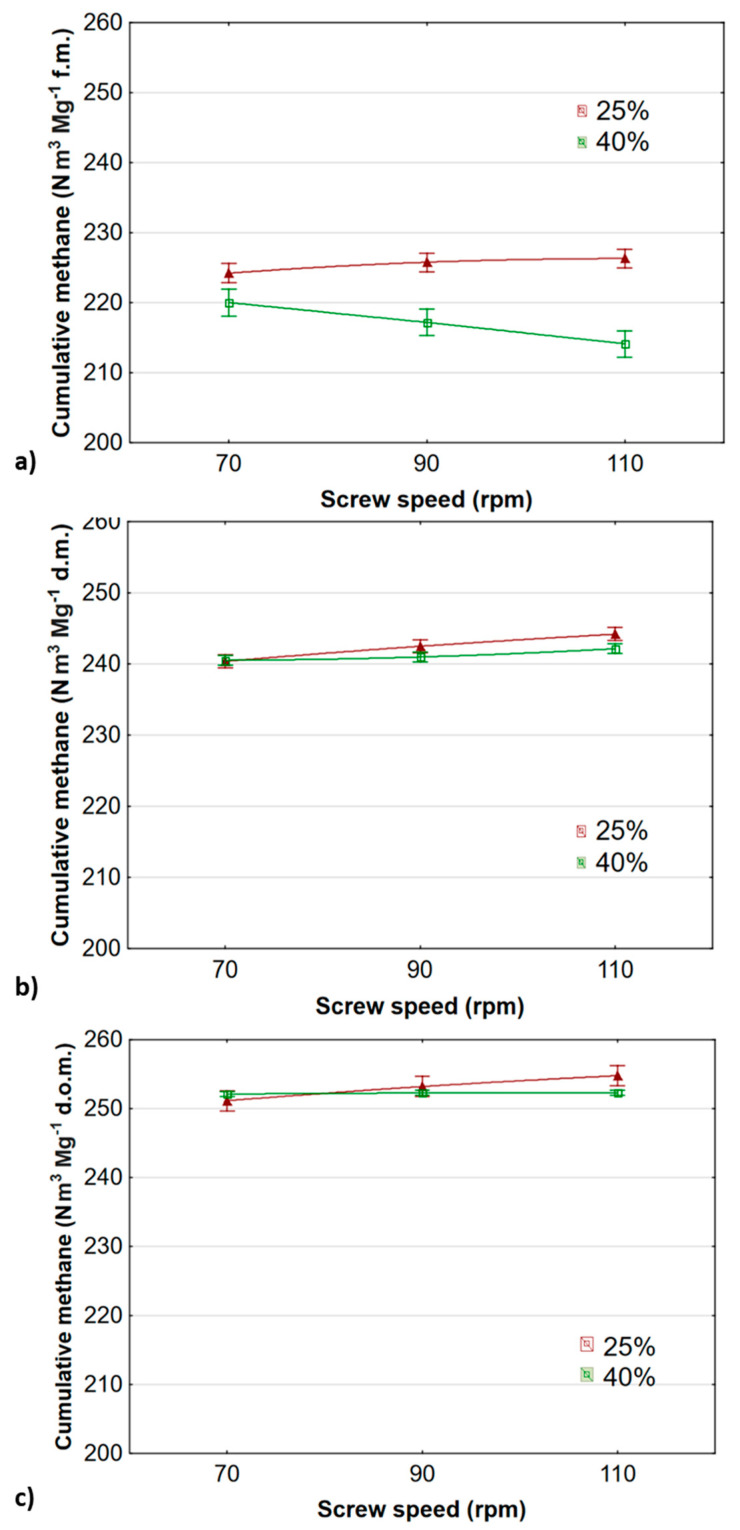
Cumulative methane efficiency from corn straw subjected to extrusion treatment at various screw speed and moisture content: (**a**) cumulative methane for fresh mass, (**b**) cumulative methane for dry matter, (**c**) cumulative methane for dry organic matter.

In all tested samples, cumulative methane treated with extrusion-cooking was higher than in the control corn straw, and the formation of accumulated methane was more intensive, both for fresh and dry matter (Figure 4). The amount of cumulative methane production during fermentation of untreated corn straw was lower; the corresponding values were 197.34 Nm^3^ Mg^−1^ for fresh matter, 219.35 Nm^3^ Mg^−1^ for dry matter, and 228.78 Nm^3^ Mg^−1^ for dry organic matter. Extrusion-cooking pretreatment of corn straw allowed the achievement of more efficient methane efficiency with an increase of 8–15% for fresh matter, 9–11% for dry matter, and 10–11% for dry organic matter, respectively compared to untreated corn straw. Higher values of cumulative methane were obtained for dry organic matter for both moisture levels used in the experiment compared to other efficiency calculations. The highest value (254.83 Nm^3^ Mg^−1^) was observed for dry organic matter for samples extruded at 25% of the initial moisture of straw and screw speed of 110 rpm (Figure 4c). The lowest value (214.06 Nm^3^ Mg^−1^) was observed for the cumulative amount of methane in fresh matter when the samples used were extruded at 40% of the initial moisture of corn straw and at the screw speed of 110 rpm during processing. Higher screw speed applied for corn straw pretreatment enhanced methane efficiency, except for fresh matter calculated when 40% of initial moisture content applied during extrusion-cooking (Figure 4a). Only in the case of moisture, a 40% increase in the initial moisture content caused a decrease in methane efficiency. Gizińska-Górna et al. [37] reported that methane production during anaerobic digestion was the highest from common reed (108 Nm^3^ Mg^−1^ f.m. and 212.5 Nm^3^ Mg^−1^ d.m.) and the lowest from Jerusalem artichoke (66 Nm^3^ Mg^−1^ f.m.). Kowalczyk-Juśko et al. [38] found that Jerusalem artichoke treated by silage process had still some organic matter not degradable by fermentation. Methane production from 1 Mg of dry matter (187.09 Nm^3^) is almost twice lower compared with maize silage (363.41 Nm^3^). The extrusion-cooking processing of Jerusalem artichoke biomass can be very efficient and can double the methane production. The increase in methane production efficiency from extruded Jerusalem artichoke only by 50% (up to 95.1 Nm^3^ Mg^−1^) made this substrate also interesting for biogas plants which commonly, in Europe, use maize silage. Pilarski et al. [10] reported the amount of methane expressed for fresh matter increasing by 16.48% for maize silage and by as much as 35.30% for maize straw silage in comparison with non-extruded substrates. They also showed that for pretreated maize straw the cumulative methane yield increased by 26.79%. This could be the result of partial hydrolysis of cellulose and hemicellulose to monosaccharides, such as glucose, xylose, mannose, and galactose, which are easily and effectively decomposed, thus providing considerable amounts of methane [10].

**Figure 5:**
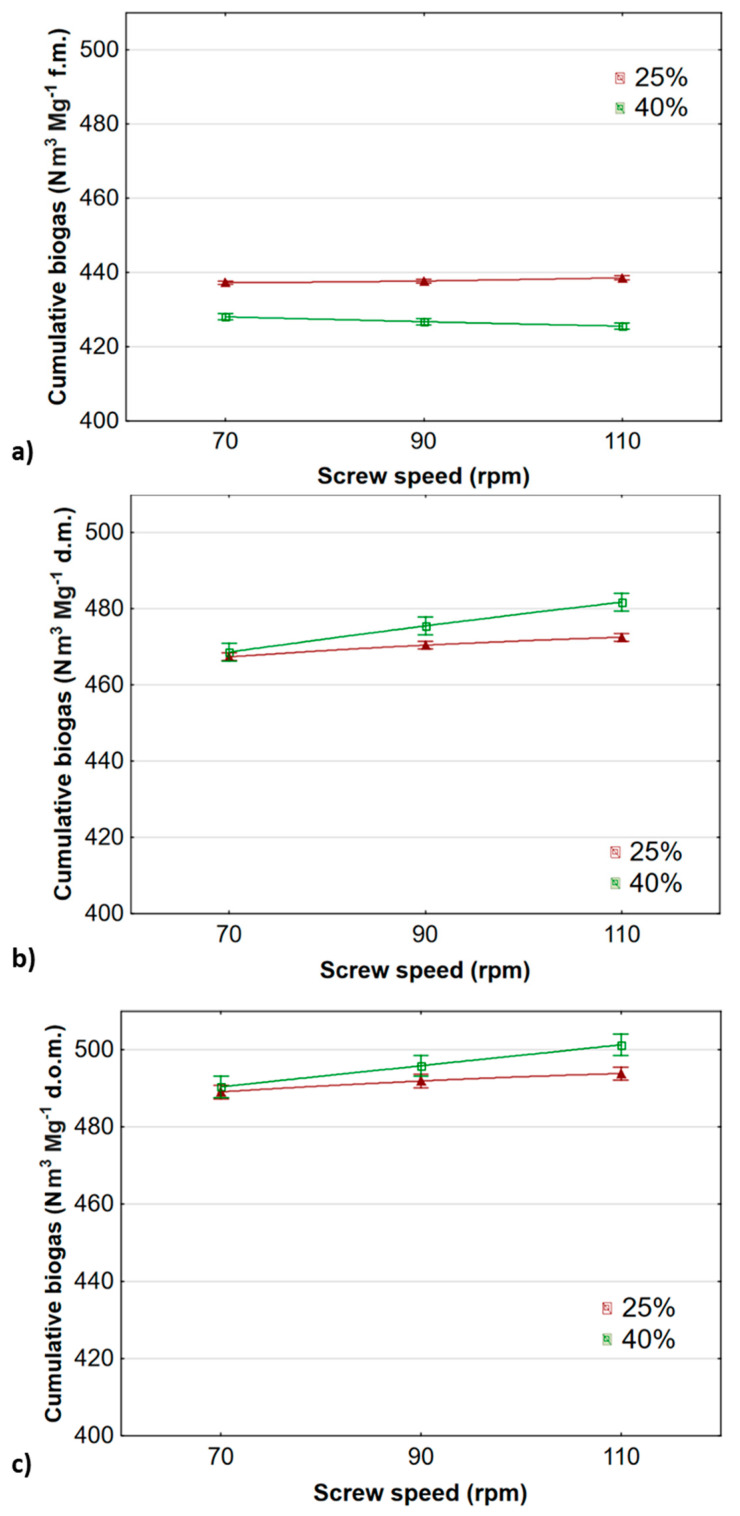
Cumulative biogas efficiency with corn straw subjected to extrusion treatment at various screw speeds and moisture content: (**a**) cumulative biogas for fresh mass, (**b**) cumulative biogas for dry matter, (**c**) cumulative biogas for dry organic matter.

The content of cumulative biogas in all tested samples was higher than in the control straw, and the formation of accumulated biogas was more intensive, both for the dry and fresh substance (Figure 5). The amount of cumulative biogas during fermentation of untreated corn straw was 398.13 Nm^3^ Mg^−1^ for fresh matter, 442.58 Nm^3^ Mg^−^^1^ for dry matter, and 461.56 Nm^3^ Mg^−1^ for dry organic matter. Extrusion-cooking pretreatment of corn straw let to increase the efficiency of biogas production during fermentation with the growth by 9–10% from substrate fresh matter, 5–7% for dry matter, and 6–7% for dry organic matter, respectively, when 25% of moisture content was used compared to untreated corn straw. For fermentation of corn straw samples pretreated at 40% of moisture content, the increase in cumulative biogas efficiency was similar. Only for samples processed at 110 rpm, it reached 14% if calculated as dry matter. The highest values of cumulative biogas were obtained for organic dry matter at both moisture levels of corn straw applied during processing. The lowest efficiency cumulative biogas (425.84 Nm^3^ Mg^−1^) was observed in samples processed at 110 rpm and 40% of initial moisture as expressed for fresh matter (Figure 5a). The highest efficiency accumulated biogas (501.98 Nm^3^ Mg^−1^) was recorded for dry organic matter samples processed at 110 rpm and at a higher moisture level of corn straw during processing (Figure 5c). Only in the case of application of 40% of initial moisture content of corn straw during processing, a slight decrease in efficiency of biogas was observed when calculated for fresh matter (Figure 5a). A significant effect (*p* < 0.05) of screw speed on cumulative biogas efficiency was observed for almost all variables, both in F-test and KW tests (Table 1). In almost all cases, high values of correlation coefficients suggest a positive effect on biogas efficiency of increased screw speed during the extrusion-cooking of corn straw. Only for cumulative methane and biogas, the effect was opposite when the initial moisture content was 40%. Kowalczyk-Juśko et al. [38] proved that biogas efficiency with maize could be at the range of 684.70 Nm^3^ Mg^−1^ d.m., i.e., much higher than biogas production from Jerusalem artichoke: 375.30 Nm^3^ Mg^−1^ d.m.

Maize straw extrusion studies were also conducted by other authors. Kozłowski et al. [39] conducted research on the energy potential of the untreated maize straw and extruded maize straw used in biogas production. Cumulated biogas for untreated maize straw was 407.81 Nm^3^ Mg^−1^ and 438.40 Nm^3^ Mg^−1^ for extruded maize straw (fresh mass) and methane production respectively by 7.50 and 8.51%. Amith Abraham et al. [40] using the extrusion process obtained a 33% increase in biogas productivity and Dell’Omo 49.1% [41].

### 3.4. Analysis of Samples Using FTIR Infrared Spectroscopy

**Figure 6:**
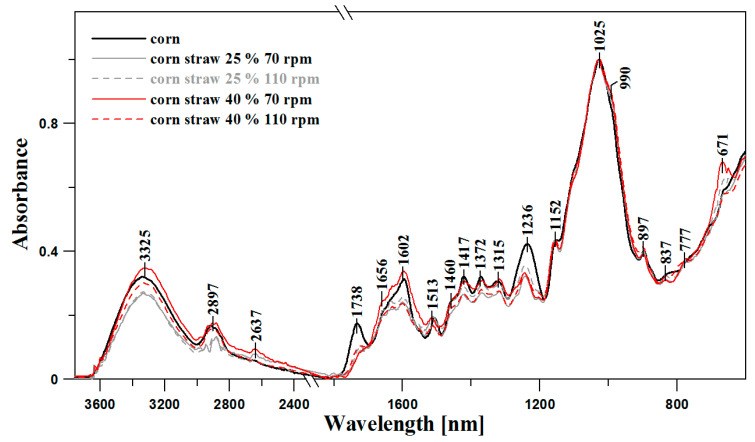
Normalized FTIR infrared spectra for samples of: corn, corn straw 25% and 70 rpm, 25% and 110 rpm, and corn straw 40% and 70 rpm and 40% and 110 rpm for the spectra of 3750–600 cm^−1^.

FTIR infrared spectroscopy was employed to characterize selected samples at the molecular level in greater detail. For more convenient presentation, description, and interpretation of obtained results, all bands are shown in Figure 6 and Table 2 (for the spectral range of 3700–650 cm^−1^). For the samples of corn, corn straw at 25% of moisture content (at 70 and 110 rpm) and for corn straw at 40% of moisture content (at 70 and 110 rpm), as shown in Table, the vibrations of characteristic functional groups found in systems with more lignocellulose structures were assigned to the appropriate bands.

As reported by Kondo [42], Liang et al. [43] or Hospodarova et al. [44], the first very characteristic vibration area in the tested samples, i.e., vibrations with a maximum at ~3325 cm^−1^, concerns tensile vibrations of -OH groups present in the lignocellulose structure, which are the main component of the samples selected for testing (Table 2 and Figure 6). Evidently (as shown in Figure 6), the intensity of vibrations from this area is mainly amplified by a change in the humidity level in the tested samples. This band, as seen in the literature data, may exhibit noticeable shifts as the connections of the components for minginter molecular hydrogen bonds increases, which may take place in this case. Another particularly important area corresponds to the tensile vibrations of C-H groups in CH_2_ and CH_3_ groups in the lignocellulose structure, the maximum of these vibrations is about 2897 cm^−1^, although the area is irregular, probably because of the used agents. These vibrations, mainly due to the variable rpm values, 70 and 110, (particularly evident in this case) differ significantly and are fairly irregular, which can indicate a higher level of degradation of samples selected for testing. Quite intensive and irregular absorption of the wide vibrations of the -OH groups described above upholds the much weaker tensile vibrations of the C-H groups in the tested structures (see Figure 6).

It is worth stressing that the usually very broad vibration bands ν(-OH) are the result of formation of strong hydrogen bonds belonging to the interactions between structural units in the main component of the tested samples, i.e., lignocellulose [42]. The deformation vibrations of -OH groups, on the other hand, correspond to the band with the maximum of ~1656 cm^−1^ [43,45]. In the case of the tested samples, this band may also come from tensile vibrations C=C [46]. Another very important vibration area concerns bands with a maximum of about 1738 cm^−1^ corresponding to the tensile vibrations of the carbonyl group (Figure 6). This area (occurring mainly as a reinforcement of the band with a maximum of about 1656 cm^−1^) differs significantly in intensity depending on the moisture content in the sample as well as screw speed during its processing. A noticeable change in the vibration intensity for this band can be a clear indication of degradation processes occurring in the tested samples.

Moving on to the fingerprint region (~1500 and 700 cm^−1^), the presented spectra reveal rich bands that are significant for interpretation reasons. The following vibrations need to be mentioned: deformation vibrations of the C-H group (1370 cm^−1^) [47], deformation vibrations of CH_2_ (1426 cm^−1^), and deformation vibrations of the –OH groups visible in the chemical structure of the main compound of the tested samples, i.e., lignocellulose [46,48]. Highly reliable are also vibrations with the maximum of ~1152 cm^−1^ originating in the tensile vibrations of the ring as well as in the tensile vibrations of the C–O groups. On the other hand, vibrations with the maximum at ~1025 and ~989 cm^−1^ mainly come from the tensile vibrations of the C-O groups. It should be noted that, despite the agents involved, these bands do not differ much compared with the spectrum of the control sample (maize) in this spectral range. These bands owe their intensity mainly to the tensile vibrations in the C-O-C group of the lignocellulose structure. At the end of the description and discussion of the obtained FTIR vibrations, small but noticeable differences should also be highlighted in the shape of the bands in the range from 900 to 650 cm^−1^. These are the vibrations in sugar fraction bonds forming the lignocellulose structure. These vibrations reveal the greatest changes in the area of ~671 cm^−1^, i.e., in the area with extra deformation vibrations of -OH groups which can be part of the formation of hydrogen bonds between individual lignocellulose units. To sum up, it should be noted that the performed FTIR spectroscopy studies offer an extensive interpretative perspective for further studies of this type of materials to be carried out in subsequent stages. The bands that are the most reliable in terms of assessment of degradation changes occurring in the analyzed samples are the vibrations described in detail above, i.e., 3325, 2897, 1738, 1236, 837, and 671 cm^−1^.

## 4. Conclusions

Pretreated corn straw moistened to 25% of initial moisture content and processed at the highest rpm during the extrusion-cooking process proved to provide the most efficient conditions for methane and biogas production efficiency. Both in the case of dry matter and dry organic matter, the rotational speed of the extruder screw had an impact on the production of biogas and methane. With a higher rotational speed of the extruder screw, the production of cumulated biogas and methane increased. These conditions showed to have the least energy demand during pretreatment, so they can be recommended as a pretreatment method useful for increasing biogas efficiency at low energy expenditure. Pretreatment of corn straw by extrusion-cooking increased the amount of methane accumulated in the fresh biomass matter as well as accumulated biogas compared to unprocessed samples. The amount of methane and biogas accumulated from extruded organic dry matter grew compared to the untreated biomass of corn. Because of low cost and availability (post-consumer waste), corn straw is the ideal raw material for use in agricultural biogas plants. Because of the difficult degradation properties of this material, it must be pre-processed. Pretreatment of corn straw showed a positive effect on the acquisition of biogas and methane. Our studies demonstrate that the energy used for pretreatment is significantly lower compared to the increase in energy associated with the increase in methane efficiency. Residues obtained after the fermentation process can be used as a fertilizer, meaning that no post-production waste is generated, and the raw material is fully used. In the FTIR studies, bands were clearly detected which indicate the influence of the applied agents on the processes, which indicates that the selection of appropriate agents may significantly accelerate the degradation of the main lignocellulose structures during the degradation processes. Further research should be based on finding optimal parameters of pretreatment of other cereal straw or energy plants that allow obtaining highest (51.63% for pre-treated straw) efficiency biogas and methane while maintaining low energy requirements.

## Figures and Tables

**Table 1:** Regression equations, correlation coefficients and statistical one-way analysis of variance (ANOVA) with F-test and Kruskal-Wallis test depend on screw speed used during pretreatment of corn straw.

Variable	Moisture Content (%)	Equation	Correlation Coefficient	F-Test	*p* Value	Kruskal-Wallis Test	*p* Value
SME (kWh kg^−1^)	25	0.463 − 0.108x + 0.014x^2^	−0.974	105.315	0.00002 *	7.200	0.0273 *
	40	0.232 − 0.021x + 0.003x^2^	−0.919	18.611	0.0027 *	7.200	0.0273 *
WAI (g g^−1^)	25	4.243 + 0.012x + 0.024x^2^	0.717	3.282	0.1089	3.518	0.1722
	40	6.181 − 0.379x + 0.003x^2^	−0.696	2.823	0.1367	5.535	0.0628
WSI (%)	25	3.295 − 0.524x + 0.078x^2^	−0.312	0.339	0.7253	1.412	0.4937
	40	1.134 + 0.694x − 0.019x^2^	0.838	7.055	0.0266 *	6.489	0.0390 *
Methane content (%)	25	51.24 + 0.145x − 0.005x^2^	0.982	82.765	0.00004 *	7.200	0.0273 *
	40	52.47 − 1.282x + 0.182x^2^	−0.715	3.381	0.1039	4.622	0.0992
Cumulative methane (Nm^3^ Mg^−1^ f.m.)	25	221.66 + 3.082x − 0.512x^2^	0.816	7.687	0.0221 *	6.879	0.0321 *
	40	222.6 − 2.487x − 0.113x^2^	−0.952	28.984	0.0008 *	7.200	0.2730
Cumulative biogas (Nm^3^ Mg^−1^ f.m.)	25	437.21 − 0.138x + 0.202x^2^	0.924	21.939	0.0017 *	6.879	0.0321 *
	40	429.54 − 1.52x + 0.067x^2^	−0.940	22.785	0.0016 *	7.2605	0.0265 *
Cumulative methane (Nm^3^ Mg^−1^ d.m.)	25	237.81 + 2.755x − 0.208x^2^	0.970	51.939	0.0002 *	7.200	0.2730
	40	240.74 − 0.583x + 0.35x^2^	0.895	17.006	0.0034 *	6.252	0.0439 *
Cumulative biogas (Nm^3^ Mg^−1^ d.m.)	25	448.33 + 27.082 − 8.005x^2^	−0.296	0.588	0.5843	2.400	0.3012
	40	461.07 + 7.892x − 0.332x^2^	0.984	91.690	0.00003 *	7.200	0.2730
Cumulative methane (Nm^3^ Mg^−1^ d.o.m.)	25	248.59 + 2.77x − 0.237x^2^	0.924	18.196	0.0028 *	7.200	0.0273 *
	40	251.84 + 0.352x − 0.068x^2^	0.383	0.675	0.5439	1.367	0.5049
Cumulative biogas (Nm^3^ Mg^−1^ d.o.m.)	25	485.31 + 4.258x − 0.468x^2^	0.933	22.458	0.0016 *	7.200	0.0273 *
	40	484.95 + 5.53x − 0.023x^2^	0.970	47.510	0.0002 *	7.200	0.0273 *

*—indicates significant effect; x—screw speed; f.m.—fresh matter; d.m.—dry matter; d.o.m.—dry organic matter.

**Table 2:** The location of the maxima of FTIR absorption bands with the arrangement of appropriate vibration for selected samples of: corn, corn straw 25% and 70 rpm, 25% and 110 rpm and corn straw 40% and 70 rpm and 40% and 110 rpm for the spectra of 3750–600 cm^−1^.

FTIR					Type and Origin of Vibrations
Position of Bands [cm^−1^]					
Corn	Corn Straw 25% 70 rpm	Corn Straw 25% 110 rpm	Corn Straw 40% 70 rpm	Corn Straw 40% 110 rpm	
3332	3332	3336	3320	3324	ν (O-H) in H_2_O And intra-molecular hydrogen bonding
2912	2936	2944	2924	2912	ν (C-H) in CH_2_ and CH_3_ asymetrical and symmetrical
2876	2892	2892	2888	2884	
1731	1727	1725	1724	1731	ν (C=O)
1656	1663	1661	1660	1665	ν (C=C)and δ (O-H)
-	1635	1639	1626	1633	δ (O-H) adsorbed H_2_O
1599	1597	1599	1597	1603	ν (C=C)
1509	1511	1516	1513	1513	
1460	1460	1462	1458	1460	δ (-OH in plane), δ (CH_2_), δ (C-H)
1424	1422	1422	1426	1426	
1373	1375	1368	1371	1371	
1322	1319	1320	1317	1320	δ (C-H) and δ (O-H)
1236	1239	1241	1249	1239	δ (C-H) and antisymmetrical bridge oxygen stretching –OH in-plane bending
1158	1156	1156	1160	1158	antisymmetrical in phase ring stretching and ν (C-O)
1096	1098	1100	1106	1096	
1028	1025	1026	1028	1030	
987	994	994	989	989	ν (C-O)
896	895	896	895	898	CH_2_ rocking β-linkage of cellulose ring breathing and antisymmetrical out of phase stretching -OH out-of-plane bending
827	832	834	834	836	
778	782	774	774	778	
702	-	-	-	-	
663	659	663	669	659	

ν—stretching vibrations, δ—deformation vibrations

## Abbreviations

List of symbols and abbreviations:

rpm	rotations per minute
Nm^3^ Mg^−1^	normal cubic meter per megagram
FTIR	Fourier transform Infrared spectroscopy
NaWaRo	NachWachsendeRohstoffe
L/D	length/diameter
SME	specific mechanical energy (kWh kg^−1^)
n	screw rotation (rpm)
nm	screw rating rotation (rpm)
O	engine load compared to maximum (%)
P	rated power (kW)
Q	process efficiency (kg h^−1^)
WAI	water absorption index (g g^−1^)
WSI	water solubility index (%)
ATR	Attenuated Total Reflection
SD	standard deviation
f.m.	fresh matter
d.m.	dry matter
d.o.m.	dry organic matter
ATR	Attenuated Total Reflection
